# Surgery.-Genito-Urinary

**Published:** 1904-05-21

**Authors:** 


### PROGRESS IN MEDICINE AND SURGERY.

### SURGERY.—GENITO-URINARY.

Senile Enlargement of the Prostate.—C. S. Wallace [1] has discussed the anatomy of the gland in relation to the procedure known as total prostatectomy. In the normal prostate there is no division into lobes, the circumferential portion is fibromuscular and devoid of gland tissue, and may be called the cortex (Shattock). The gland tissue is most marked at the sides of and behind the urethra. Immediately in front of the urethra, the so-called anterior commissure is devoid of glandular tissue. Further, outside the cortex lies the fibrous sheath derived from the recto-vesical fascia, and in this is the venous plexus. This sheath is not a definite structure like the fascia lata but consists of areolar planes, and it forms the boundary of the cavity left if the prostate be removed entirely. In a specimen of enlarged prostate, obtained after death, adenomatous masses had been shelled out from both lateral parts, leaving a bilocular cavity. Then it was found that the wall of the bilocular cavity could be shelled out leaving a unilocular cavity. The wall of the latter contained glandular tissue and must have been derived from the glandular part of the organ. Outside this wall lay the prostatic plexus of veins. Thus it appeared that the adenomata in their growth stretched the prostatic tissue over themselves and forced the fibres of the prostatic tissue to take a circumferential direction and form a definite envelope for them. It is to be concluded, therefore, that in the so-called operation of total prostatectomy an envelope of expanded prostatic tissue is left behind. The adenomata may surround the urethra or form masses in the lateral lobes. In the above operation the recto-vesical fascia is not opened, and the plexus of veins is not therefore injured. Sir Thornley Stoker has discussed suprapubic prostatectomy.[2] Having opened the bladder, the prostate should first be carefully explored to determine whether one or both sides of the gland require removal, or whether a myomatous outgrowth may alone need removal. If the prostate is essentially enlarged, or if a nodular condition of the gland exists due to separate encapsuled tumours, then the entire gland should be removed, for if a partial operation only be done trouble may recur. The writer does not believe in the existence of the so-called middle lobe, the structure so named being in reality an outgrowth of a separate adenoma or myoma. The next step is to snip through the mucous membrane of the floor of the bladder on one side of the middle line, viz., over the most projecting part of the larger lobe. Scissors are used for this purpose guided by the left index finger. The incision need not be more than an inch long. Through this the right forefinger is introduced and the enucleation proceeded with as rapidly as possible. Ten to twenty minutes suffice for this. Records seem to show that the cases do just as well even if the prostatic urethra come away. The author attaches importance to the application of a thick silk suture on each side, through the bladder wall near the upper end of the incision and through all the structures of the abdominal wall except the skin. It keeps the peritoneal pouch above the bladder cicatrix. The suture is removed on the fourth or fifth day. No other sutures are advised to be used. The author considers that there are three types of senile enlargement of the prostate, viz.: (1) A true hypertrophy; (2) a condition in which the enlargement is due to encapsuled myomata or adenomata in the gland, and (3) a mixed condition of hypertrophy and interstitial growths. G. Barling[3] has reported ten cases of suprapubic prostatectomy and discussed the technique. He usually introduces a Fergusson speculum after opening the bladder and visually examines the prostatic outgrowth. Often enormous venous dilatation in the mucous membrane over the gland is found. During the operation a good-sized metal bougie is retained in the urethra. In the majority of cases the prostatic urethra comes away and the membranous urethra may be torn away also. The patient is allowed up usually in the third week after the operation. Three of the ten patients died: one of malignant disease of the colon during convalescence, one from uræmia in the third week, and one from angina pectoris in the sixth week. Stricture from cicatrisation has not followed the operation, and the results have been far beyond those attained by the operations previously in vogue. O. Horwitz[4] read a paper on the radical operations for enlarged prostate at the New York Academy of Medicine recently. Of all the methods which had been advocated, but two stood the test of experience, the Bottini operation and prostatectomy. He had previously tabulated the results of the Bottini operation, viz., 896 operations done by 48 operators, with 84·3 per cent. cured or improved, 10 per cent. unimproved, and with a mortality of 5·7 per cent. In his opinion the operation would always be regarded as a valuable surgical procedure capable of giving relief in certain cases without special danger to life. The author had performed the Bottini operation 93 times, suprapubic prostatectomy 9 times, and complete perineal prostatectomy 34 times, and he regarded the perineal operation as the best. The mortality, in experienced hands, would not be over 5 to 7 per cent. In 90 per cent. of the cases the prostate could be reached by the median perineal incision. G. E. Goodbody (San Francisco)[5] reports that he has operated by the median perineal route for hypertrophy of the prostate 73 times without any immediate mortality. All the operations were performed under spinal anæsthesia. He now uses a small incision, usually about an inch in length, in front of the central point of the perineum. The needle for cocaine injection is inserted between the second and third lumbar vertebræ. J. E. Moore reports that he has seen impotence and incontinence of urine or dribbling follow prostatectomy. Occasionally a recto-vesical fistula is a result. Epididymitis is quite a common sequel, sometimes becoming so troublesome that the testicle has to be removed. B. G. H. Moynihan[6] reports that he has operated on 12 cases of senile hypertrophy of the prostate by removing the prostate and prostatic urethra by the suprapubic method, with one fatality. The following are points in the author's technique. The bladder is washed out thoroughly with, and afterwards filled with 1 per cent. carbolic acid solution. The right first and second fingers are passed into the rectum and a rubber glove is worn over the right hand until the rectal proceedings are finished. The bladder on each side of the incision is fixed to the parietes by a silk-worm gut suture passed through its walls and the whole thickness of the abdominal wall. The small opening made into the mucous membrane is torn larger until it completely encircles the internal meatus and the anterior portion of the prostate urethra at its junction with the membranous portion is torn through after the prostate is elsewhere entirely free. At the end of the fourth day the tube used for drainage of the bladder is removed and the patient allowed to sit up with a bed rest. On the fourth and each succeeding day a catheter is passed and the bladder is freely washed with dilute carbolic lotion. On the seventh day the catheter is tied in and a drag placed upon the suprapubic wound. If the patient is feeble he may have the catheter removed and be allowed up on alternate days.

[1] Lancet, Dec. 5, 1903, p. 1578. [2] Brit. Med. Jour., Jan. 30, 1904, p. 229. [3] Ibid., p. 232. [4] Med. Rec., Jan. 15, 1904, p. 112. [5] Ibid., Jan. 23, 1904, p. 157. [6] Annals of Surgery, Jan. 1904, p. 1.

